# Association of Knowledge of HIV and Other Factors with Individuals’ Attitudes toward HIV Infection: A National Cross-Sectional Survey among the Japanese Non-Medical Working Population

**DOI:** 10.1371/journal.pone.0068495

**Published:** 2013-07-16

**Authors:** Guoqin Wang, Koji Wada, Keika Hoshi, Nanae Sasaki, Satoshi Ezoe, Toshihiko Satoh

**Affiliations:** 1 Kitasato Clinical Research Center, Kitasato University School of Medicine, Sagamihara, Kanagawa, Japan; 2 Department of Public Health, Kitasato University School of Medicine, Sagamihara, Kanagawa, Japan; 3 Department of Preventive Medicine, Kitasato University School of Medicine, Sagamihara, Kanagawa, Japan; 4 Komatsu Ltd Health Promotion Center, Shonan Area, Hiratsuka, Kanagawa, Japan; 5 The Joint United Nations Programme on HIV and AIDS (UNAIDS), Geneva, Switzerland; State University of New York, Oswego, United States of America

## Abstract

**Background:**

The stigma of and discrimination because of HIV has been described as the most important obstacle to prevention and treatment efforts. The purpose of this study was to investigate negative attitudes and prejudice toward HIV among the Japanese non-medical working population and to explore contributing factors.

**Methods:**

An online anonymous nationwide survey involving approximately 3,000 individuals was conducted in Japan. Questions ranged from background information and HIV knowledge to individuals’ attitudes towards HIV infection in the workplace. Descriptive statistics and logistic regression were applied for analysis.

**Results:**

Thirty-three percent of participants feared transmission of HIV from infected colleagues, 34% tended to avoid contact with them and 40% had prejudiced opinions about HIV infection. Despite a relatively high level of knowledge of HIV/AIDS overall (11.9±3.3 from 15 points), only 50% of individuals were aware of some issues. Greater knowledge was associated with less negative attitudes towards HIV infection (OR 0.39, 95% CI 0.31–0.48 for prejudiced opinion, high compared with low level of knowledge), whereas greater health consciousness was inversely related to attitude (OR 1.97, 95% CI 1.50–2.58 for prejudiced opinion, high compared with low health consciousness).

**Conclusion:**

Knowledge neutralizes peoples’ negative attitudes towards HIV infection, whereas greater health consciousness may worsen them. Educational programs should balance knowledge with health consciousness to improve the efficacy of HIV interventions.

## Introduction

Despite the fact that the risk of transmission of HIV through contact is very low, feelings of fear and avoidance of infected people have prevailed since the discovery of the infection [1]. HIV-related stigmas–defined as prejudice, discounting, discrediting and discrimination directed at people with HIV/AIDS [2]–deter individuals from undergoing HIV testing, cause HIV positive people to avoid disclosure and subsequently lead to delays in them receiving optimal care; stigmas have thus been described as barriers to HIV prevention and treatment efforts [3].

Although the stigma of HIV has been well documented, most studies have focused on high risk populations or health providers [2], [4], [5], [6], [7]; there is a lack of investigations of the attitudes of general individuals in the work environment toward colleagues infected with HIV [8]. This is possibly because, from the scholar’s perspective, the workplace is unlikely to be a high risk location for HIV transmission. However, even among the general work environment, negative attitudes toward people with HIV may lead to stigma and discrimination. Negative attitudes are supposed to be associated with levels of knowledge about HIV/AIDS, and lower levels of knowledge and misconceptions about HIV may lead to more negative attitudes. Evidence indicates that individuals infected with HIV may face isolation and discrimination by their colleagues and encounter difficulties in the workplace even when they can contribute effectively to a company [9], [10], [11]. Unemployment rates among people with HIV are reported to be 45% or higher [12], [13], and unemployment and low social status in HIV infected individuals may lead to a worse prognosis, including reduced survival, even in those receiving antiretroviral therapy [14]. Although, these may mainly reflect situations of developing countries, it implies the impact of negative opinions on HIV/AIDS.

In Japan, the number of reported cases of HIV and AIDS continues to increase [17]. According to the report to UNAIDS, a total of 12,648 cases of HIV infection and 5,799 AIDS were reported as of the end of 2010. The majority of individuals infected with HIV are men who have sex with men (MSM) [15], [16], which accounts for more than 50% of the reported cases. Approximately, 70% of newly reported cases were in their twenties or thirties. These aspects highlight the necessity for improved early detection and prompt treatment. However, barriers to these efforts, including negative stereotypes of HIV infected people, have not been investigated. To facilitate the planning and implementation of more effective intervention programs, it is necessary to determine attitudes towards HIV among the general working population in Japan. In this study, we aimed to: (1) investigate negative attitudes and prejudiced opinions about HIV among the Japanese non-medical working population; and (2) determine whether level of knowledge about HIV and other factors may contribute to people’s attitudes towards HIV.

## Methods

### Ethics Statement

This survey was approved by the institutional ethics committee of the Kitasato University School of Medicine. Response to the questionnaire was taken as agreement to participate in the study.

### Participants and Conduct of the Survey

An online, anonymous, self-administered questionnaire was sent to individuals from 47 prefectures in 10 areas of Japan. Participants were selected randomly from members having registered with a commercial survey company, using a stratified sampling method with gender and age. The gender composition was 1∶1 and the numbers of participants in each age group were equal. Financial incentives for each participant valued about a few US dollar. To reflect the non-medical working population, members who registered as doctors, nurses and other medical staff were excluded from participation.

The cross-sectional survey comprised 24 questions ranging from the participants’ demographics (four items) to knowledge of essential factors concerning HIV transmission (15 items) and general attitudes toward HIV infection (three items), accompanied by one question about the participants’ health consciousness and one question about their experience of HIV testing. Patients’ background information comprised gender, age, educational level and occupation. Educational levels were divided into: graduation from high school or below; technical college; and college or higher. Occupations included regular company employees, managers, other professionals such as teachers or lawyers, temporary or part-time workers, freelance or retired (unemployed) workers, homemakers and graduate students. The one question about the participants’ health consciousness was defined as “To what extent do you think you have health consciousness?” followed by an answer of “low, moderate, high or very high”; and the question about patient’s experience of HIV testing was simply defined as “ Have you ever received a HIV testing?” followed by a “yes” or “no” answer.

Knowledge about HIV transmission was investigated with 15 statements based on outlines provided by UNAIDS (2003) [18]. The statements were grouped into four domains: domain 1 was related to misconceptions about HIV transmission via talking, shaking hands or sharing a living space (three items); domain 2 was related to misconceptions about HIV transmission via sharing dishes/food, hot springs, kissing as a greeting or mosquito bites (four items); domain 3 was related to the risk of HIV transmission via sexual acts, blood transfusion, or by sharing syringes for injecting drug use (four items); and domain 4 was related to knowledge about carrying HIV in a healthy-looking person (four items). A “True” or “False” option was offered for each statement and each correct answer accounted for 1 point from a total score of 15 points. The total score was classified as low, moderate or high and was used for the analysis together with the scores for each domain of HIV knowledge.

We used the following attitudes toward HIV infection as the outcome variables of the study: (1) participant would worry about transmission if a colleague were infected; (2) participant would avoid contact with an infected person; and (3) participant had prejudiced opinions about infected persons. A four-point Likert scale assessment was used for each opinion (1 = strongly disagree to 4 = strongly agree); the answers “agree” and “strongly agree” were considered negative opinions and used for logistic regression analyses.

### Statistical Analysis

Continuous parameters were presented as the mean ± s.d. and categorical data were given as proportions of the total sample. To explore associations between participants’ attitudes toward HIV and their knowledge about HIV and other factors, each statement of opinion was treated as a separate outcome. Univariable and multivariable logistic regressions were applied; factors included in the multivariable analyses were gender, age, educational level, occupation, knowledge about HIV, experience of HIV testing and health consciousness. With respect to knowledge about HIV, the total score or the score for each domain was entered into each model. We assumed that 30% of the general population holds negative opinions towards HIV. To detect a 5% difference in the proportions of patients' attitudes between 2 groups, at an alpha level of 0.005, a sample size of 3000 was assumed to give a power of more than 85%. The alpha level was determined by taking potential overestimation into account with Bonferroni correction for multiple comparisons. With the target sample size of 3000, we decided the number of participants in each gender and age group. Statistical analyses were performed using Stata software, version 11.0 (StataCorp, College Station, Texas, USA). A two-tailed *P*-value of <0.005 was considered to be significant unless otherwise indicated.

## Results

### Participant Demographics and Attitudes Towards HIV Infection

The survey was sent to 7,937 individuals and closed after each stratum of gender and age group reached its target sample size. Totally, 3,055 participants had responded, of them, 1,523 (49.9%) were female and each age group (20–29, 30–39, 40–49, 50–59 and 60–69 years) contained approximately 20% of the total number of participants. The participants’ educational levels and occupations are shown in Table 1. Of the total number of participants, 24.4% and 61.0% had very high or high health consciousness and 417 (13.7%) had undergone a HIV test.

**Table 1:** Baseline characteristics of participants.

	No. (%)
	(*n* = 3,055)
**Gender**	
Female	1,523 (49.9%)
**Age (years)**	
20–29	607 (19.9%)
30–39	611 (20.0%)
40–49	612 (20.0%)
50–59	616 (20.2%)
60–69	609 (19.9%)
**Education**	
High school	878 (28.7%)
Technical college	767 (25.1%)
College	1,368 (44.8%)
Missing	42 (1.4%)
**Occupation**	
Company employee	661(21.6%)
Manager	244 (8.0%)
Employee in other profession	212 (6.9%)
Part-time worker	522 (17.1%)
Unemployed	566 (18.5%)
Homemaker	649 (21.2%)
Undergraduate	201 (6.6%)
**Health consciousness**	
Very high	746 (24.4%)
High	1,862 (61.0%)
Low	447 (14.6%)
**History of HIV testing**	
Yes	417 (13.7%)
No	2,621 (85.8%)
Missing	17 (0.96%)
**Attitudes towards HIV infection in the workplace**	
Worry about transmission	
Strongly agree	250 (8.2%)
Agree	762 (24.9%)
Disagree	990 (32.4%)
Strongly disagree	891 (29.2%)
Missing	162 (5.3%)
Avoid contact with infected colleague	
Strongly agree	238 (7.8%)
Agree	811 (26.6%)
Disagree	953 (31.2%)
Strongly disagree	849 (27.8%)
Missing	204 (6.7%)
Prejudiced opinion about HIV infection	
Strongly agree	222 (7.3%)
Agree	993 (32.5%)
Disagree	910 (29.8%)
Strongly disagree	700 (22.9%)
Missing	230 (7.5%)

In terms of the participants’ attitudes towards HIV infection, 250 (8.2%) and 762 (24.9%) strongly agreed or agreed with the statement that they would worry about transmission if a colleague were infected. One thousand and forty-nine (34.4%) would avoid contact with an infected colleagues; among these, 238 (7.8%) strongly agreed with this statement. Two hundred and twenty-two (7.3%) and 993 (32.5%) participants strongly agreed or agreed that they would have prejudiced opinions about an HIV infected colleague, representing 39.8% of the total.

### Individuals’ Levels of Knowledge about HIV

Knowledge about HIV transmission via talking, shaking hands, sharing a living space, sexual acts, blood or sharing syringes for drug use was high; more than 90% of answers were correct concerning statements in these domains. However, the percentages of correct answers concerning HIV transmission via sharing dishes/food, hot springs, kissing as a greeting or mosquito bites varied from 50% to less than 80%. The lowest levels of knowledge concerned HIV transmission via kissing as a greeting (51.5%) or by mosquito bites (56.0%) and the possible presence of unapparent HIV infection in a healthy-looking person (58.4%).

The mean total score for the participants’ HIV knowledge was 11.9±3.3 (Table 2). One thousand and eighteen participants (33.3%) scored less than the mean; this group was defined as having a low level of knowledge. Seven hundred and forty-one (24.3%) answered all 15 statements correctly and were defined as having a high level of knowledge. The remaining 1,296 participants (42.5%) scored 12–14 and were defined as having a moderate level of knowledge. The internal validity among statements in each domain was satisfactory (Cronbach’s alpha 0.75–0.95) and the scores for each domain of HIV knowledge were 2.8±0.7, 2.6±1.4, 3.7±0.8 and 2.9±1.4, respectively (Table 2).

**Table 2:** Basic knowledge related to HIV infection.

	No. (%)	Cronbach’s alpha	No. (%) or mean ± s.d.
			(*n* = 3,055)
**Current HIV knowledge (15 items)**			
Total score	3,055 (100%)	0.89	11.9±3.3
Low (1–11 points)	1,018 (33.3%)		
Middle (12–14)	1,296 (42.5%)		
High (15)	741 (24.3%)		
Domain 1 (three items)			
Risk of HIV transmission via talking, shaking hands or sharing living space	3,055 (100%)	0.95	2.8±0.7
Domain 2 (four items)			
Risk of HIV transmission via sharing dishes, hot springs, kissing as greeting or mosquito bite	3,055 (100%)	0.76	2.6±1.4
Domain 3 (four items)			
Risk of HIV transmission via sexual acts, blood or sharing syringes	3,055 (100%)	0.75	3.7±0.8
Domain 4 (four items)			
Knowledge of unapparent infection with HIV	3,055 (100%)	0.82	2.9±1.4

### Association between HIV Knowledge and other Factors and Participants’ Attitudes Toward HIV Infection

On univariable analysis, HIV knowledge and high health consciousness were found to be associated with worrying about transmission of HIV (Table 3), avoiding contact with infected colleagues (Table 4) and having prejudiced opinions about infected colleagues (Table 5). These associations remained after adjusting for potential confounders by multivariable analysis. The higher the level of knowledge, the lower the proportion of participants with negative attitudes (OR 0.19, 95% CI 0.15−0.24 for fear of transmission, Table 3; OR 0.18, 95% CI 0.14−0.23 for avoidance of contact, Table 4; OR 0.39. 95% CI 0.31−0.48 for prejudiced opinion, Table 5; high vs low level of knowledge). The situation for health consciousness was reversed; greater health consciousness was related to a higher proportion of participants having negative attitudes (OR 2.41, 95% CI 1.80−3.22 for fear of transmission; OR 2.24, 95% CI 1.68−2.98 for avoidance of contact; OR 1.97, 95% CI 1.50−2.58 for prejudiced opinion; very high vs low health consciousness).

**Table 3:** Factors associated with attitude towards HIV infection: worry about transmission.

								Univariablemodel								Multivariablemodel								
								OR					95% CI	*P*		OR					95% CI	*P*		
**Gender**																								
				Female				1.04				0.90–1.22		0.59		1.03				0.83–1.27			0.79	
**Age**																								
				20–29				ref.								ref.								
				30–39				1.05			0.83–1.35			0.65		1.13			0.85–1.50					0.41
				40–49				0.94			0.74–1.20			0.63		0.97			0.73–1.30					0.86
				50–59				1.07			0.84–1.37			0.56		1.08			0.81–1.45					0.58
				60–69				1.11			0.87–1.41			0.41		1.05			0.78–1.43					0.73
**Education**																								
			High school				ref.									ref.								
			Technical college				0.93			0.76–1.16				0.54		0.89			0.71–1.11					0.29
			Above college				1.02			0.85–1.23				0.82		1.05			0.85–1.29					0.64
**HIV knowledge (total 15 points)**																								
		Low (1–11)				ref.										ref.								
		Middle (12–14)				0.47			0.39–0.56						<0.001	0.45		0.38–0.54						<0.001
		High (15)				0.21			0.16–0.26						<0.001	0.19		0.15–0.24						<0.001
**Health consciousness**																								
	Low				ref.											ref.								
	High				1.55				1.21–1.98						0.001	1.7	1.31–2.21							<0.001
	Very high				2.04				1.56–2.68						<0.001	2.41	1.80–3.22							<0.001

Notes: OR = odds ratio for each factor; OR in multivariable models was adjusted for gender, age, education, occupation, HIV knowledge, health consciousness and history of HIV testing; ref. = reference.

**Table 4:** Factors associated with attitude toward HIV infection: avoid contact with infected colleague.

			Univariable model			Multivariable model		
			OR	95% CI	*P*	OR	95% CI	*P*
**Gender**								
	Female		1.00	0.87–1.18	0.9	1.01	0.82–1.25	0.91
**Age**								
	20–29		ref.			ref.		
	30–39		1.20	0.94–1.54	0.13	1.34	1.00–1.79	0.05
	40–49		1.11	0.86–1.42	0.42	1.17	0.87–1.57	0.31
	50–59		1.35	1.06–1.72	0.016	1.38	1.03–1.86	0.03
	60–69		1.61	1.26–2.05	<0.001	1.56	1.15–2.11	0.004
**Education**								
	High school		ref.			ref.		
	Technical college		0.94	0.76–1.16	0.54	0.94	0.75–1.18	0.59
	College		1.08	0.90–1.29	0.4	1.22	0.99–1.50	0.06
**HIV knowledge (total 15 points)**								
	Low (1–11)		ref.			ref.		
	Middle (12–14)		0.48	0.40–0.57	<0.001	0.47	0.39–0.56	<0.001
	High (15)		0.2	0.16–0.25	<0.001	0.18	0.14–0.23	<0.001
**Health consciousness**								
	Low		ref.			ref.		
	High		1.35	1.06–1.17	0.014	1.42	1.10–1.84	0.007
	Very high		1.92	1.48–2.51	<0.001	2.24	1.68–2.98	<0.001

Notes: OR = odds ratio for each factor; OR in multivariable model was adjusted for gender, age, education, occupation, HIV knowledge, health consciousness and history of HIV testing; ref. = reference.

**Table 5:** Factors associated with attitude toward HIV infection: prejudiced opinion about HIV infection.

		Univariablemodel					Multivariablemodel		
		OR		95% CI	*P*		OR	95% CI	*P*
**Gender**									
	Female	0.90		0.77–1.04		0.16	0.81	0.66–0.99	0.047
**Age**									
	20–29	ref.					ref.		
	30–39	1.08		0.85–1.37		0.51	1.00	0.76–1.32	0.99
	40–49	1.21		0.95–1.54		0.12	1.14	0.87–1.51	0.34
	50–59	1.32		1.04–1.68		0.021	1.22	0.92–1.62	0.16
	60–69	1.65		1.30–2.10		<0.001	1.44	1.08–1.92	0.013
**Education**									
	High school	ref.					ref.		
	Technicalcollege	0.96		0.79–1.18		0.72	0.99	0.80–1.23	0.96
	College	0.98		0.82–1.18		0.86	1.05	0.86–1.28	0.63
**HIV knowledge** **(total 15 points)**									
	Low (1–11)	ref.					ref.		
	Middle (12–14)	0.71	0.60–0.82			<0.001	0.70	0.59–0.84	<0.001
	High (15)	0.40	0.33–0.49			<0.001	0.39	0.31–0.48	<0.001
**Health** **consciousness**									
	Low	ref.					ref.		
	High	1.33	1.06–1.72		0.014		1.36	1.07–1.73	0.012
	Very high	1.83	1.42–2.37		<0.001		1.97	1.50–2.58	<0.001

Notes: OR = odds ratio for each factor; OR in multivariable model was adjusted for gender, age, education, occupation, HIV knowledge, health consciousness and history of HIV testing; ref. = reference.

Regarding the participants’ age, compared with those in their twenties, participants in their sixties were more likely to have prejudiced attitudes toward HIV infection (OR 1.44, 95% CI 1.08−1.92) and had a higher tendency to avoid contact with infected individuals (OR 1.56, 95% CI 1.15−2.11). No associations were observed for gender, education, occupation or participants’ experience of HIV testing.

When extending the evaluation to each domain of HIV knowledge (Table 6), higher scores on almost all items were associated with less negative opinions (all *P*<0.001). The only exceptions concerned HIV transmission routes (e.g. sexual acts, sharing syringes for injecting drug use), higher scores that were associated with lower percentages of fear of transmission and avoidance of contact with infected persons; however, knowledge in this domain was unrelated to having prejudiced opinions about HIV.

**Table 6:** Univariable and multivariable analyses of association between each domain of HIV knowledge and attitudes toward HIV infection.

		Univariable model			Multivariable model		
		OR	95% CI	*P*	OR	95% CI	*P*
**Worry about transmission**							
HIV knowledge	Domain 1 (3 points)	0.69	0.62–0.77	<0.001	0.66	0.59–0.75	<0.001
	Domain 2 (4 points)	0.63	0.60–0.67	<0.001	0.62	0.58–0.66	<0.001
	Domain 3 (4 points)	0.85	0.77–0.95	0.003	0.83	0.75–0.93	<0.001
	Domain 4 (4 points)	0.81	0.76–0.85	<0.001	0.79	0.75–0.84	<0.001
**Avoid contact with infected colleague**							
HIV knowledge	Domain 1 (3 points)	0.69	0.62–0.77	<0.001	0.67	0.60–0.76	<0.001
	Domain 2 (4 points)	0.62	0.59–0.66	<0.001	0.61	0.58–0.65	0.006
	Domain 3 (4 points)	0.88	0.79–0.97	<0.001	0.86	0.78–0.96	<0.001
	Domain 4 (4 points)	0.82	0.78–0.87	<0.001	0.81	0.77–0.86	<0.001
**Prejudiced opinion about HIV infection**							
HIV knowledge	Domain 1 (3 points)	0.83	0.74–0.93	0.001	0.84	0.74–0.94	0.003
	Domain 2 (4 points)	0.78	0.74–0.82	<0.001	0.78	0.74–0.82	<0.001
	Domain 3 (4 points)	0.95	0.86–1.05	0.29	0.94	0.85–1.04	0.24
	Domain 4 (4 points)	0.89	0.84–0.94	<0.001	0.88	0.83–0.93	<0.001

Notes: OR = odds ratio for each opinion; OR for each domain of HIV knowledge in the multivariable model was adjusted for gender, age, education, occupation, health consciousness and history of HIV testing; domain 1 concerns HIV transmission via talking, shaking hands or sharing living space; domain 2 concerns HIV transmission via sharing dishes/food, hot springs, kissing as a greeting or mosquito bites; domain 3 concerns HIV transmission via sexual acts, blood or sharing syringes for drug use; domain 4 indicates knowledge of unapparent infection with HIV.

## Discussion

This is the first study to investigate individuals’ attitudes towards HIV infection in the workplace among the non-medical working population in Japan. Approximately one third of participants had attitudes such as fear of transmission or would avoid an infected colleague, and an even higher percentage (40%) had prejudiced opinions. These beliefs seemed to be ameliorated by higher levels of knowledge and worsened by greater health consciousness.

There is no single, defined questionnaire for the investigation of people’s attitudes toward HIV infection. In addition, individuals’ attitudes have generally been investigated in specific populations. Among these studies, hardly any evaluation has been performed in developed countries. These factors make it difficult to compare our findings with those of other researchers. Most studies have been performed among health providers, including surgeons [19], nurses [20], dentists [21], anesthetists [22] and students at health institutes [23], [24], [25], and have generally focused on individuals’ confidence with respect to providing treatment and their willingness to care for HIV/AIDS patients [25], [26]. Studies targeting high school and college students have concentrated on their attitude toward isolating a student or family member infected with HIV from uninfected individuals [27], [28]. Specific questions such as caring for relatives with AIDS or children orphaned owing to the illness have also been investigated in African countries with a high prevalence of HIV infection. Overall, based on evidence available to us, the situation regarding individuals’ attitudes toward HIV infection is not optimistic [29], [30]. It is common but not restricted in the developing countries that HIV/AIDS has been concentrated largely among injecting drug users, sex workers and their clients, and homosexuals, and is currently incurable [31], [32]. These perceptions of HIV are thought to account for individuals’ negative attitudes and prejudiced opinions [33], [34]. Studies on the stigmatization of HIV have revealed that, among HIV uninfected individuals, such stigma manifests predominantly as prejudice, stereotyping and discrimination against people with HIV [35]. As indicated by the authors, prejudice is experienced by uninfected individuals as an emotion, such as feeling threatened by an infected individual, stereotyping is experienced as cognition and discrimination is experienced as a behavior, these interact with each other and lead individuals to distance themselves from HIV infected people.

Knowledge has been believed to be an effective tool for the prevention of HIV/AIDS [36], [37]. The results of the present study indicate a reasonably high level of HIV knowledge among non-medical working population in Japan, with the mean score being 11.9 from a total score of 15. However, knowledge about the risks of HIV transmission through public contact such as sharing food or kissing as a greeting is unsatisfactory. The situation is even worse with respect to knowledge about sharing hot springs, mosquito bites and unapparent infection, with only half of the participants answering these statements correctly. This is much lower than in Tee & Huang’s report [8], but their study involved university staff only. The belief that a healthy-looking person cannot be infected with HIV is a common misconception that can result in unprotected sexual intercourse with an infected partner. The belief that HIV is transmitted through mosquito bites may weaken people’s motivation to adopt safer sexual behavior. And the belief that HIV can be transmitted through sharing food or kissing as a greeting reinforces the stigma of HIV. Such misconceptions about HIV transmission should be addressed in Japan, despite the pre-existing high levels of HIV knowledge. This is indicated by numerous studies in which higher levels of knowledge are associated with less negative attitudes [38], [39], [40], [41], as was observed in our study. There is also an increasing body of evidence supporting the effectiveness of educational programs in improving individuals’ knowledge of and attitude toward HIV infection [42], [43]. Reinforcement of knowledge remains one of the most effective ways of improving people’s negative impressions of HIV/AIDS.

The present study also attempted to assess health consciousness as a contributor to attitudes toward HIV infection. Approximately 25% and 60% of participants showed very high or high health consciousness, respectively. Although greater health concern may lead to higher levels of knowledge, from the findings of this study it appears to be related to more negative attitudes. One explanation for this could be that the information gathered as a result of high health consciousness may not necessarily be accurate and may sometimes even worsen the situation. In addition, having a high level of knowledge does not always reflect an individual’s attitudes or actions. In our study, knowledge about the risks of HIV infection via sexual acts or injecting drug use was related to reduced fear of transmission and a lower likelihood of avoiding an infected colleague. However, such knowledge did not appear to neutralize prejudiced opinions about HIV infection. Educational programs should therefore balance knowledge with health consciousness for effective intervention.

Compared with participants in their twenties, those in their sixties were more likely to avoid infected persons and to have prejudiced opinions about HIV. Data on the HIV/AIDS epidemic in Japan show that approximately 70% of newly reported cases of HIV infection are in their twenties or thirties [17], which may account for the more favorable attitude toward HIV in these age groups. Furthermore, the participants in their sixties in our study were in their thirties when HIV was first identified in Japan, and traditional beliefs about the cause of HIV and its stereotypes may account for the more negative attitudes of the older respondents.

The present study involved a large sample of more than 3,000 participants from all regions of Japan, which is a strength. However, there are certain limitations to the study. Because it was cross-sectional, no causal relationship can be concluded from the findings. Besides, though knowledge and health consciousness were probable contributors to the participants’ attitudes toward HIV, factors influencing their level of knowledge and the mechanisms involved in health consciousness remain unknown. In addition, only some indicators of HIV knowledge and attitudes were investigated. This study was web-based, potential bias may exist due to the nature of online surveys. However, online survey have now become an acceptable method for gathering data in recent years, especially regarding population based trends and opinions – a very relevant aspect of the current study. We believe our study contributes undoubted benefits in light of the potential limitations.

This study provides the first information on attitudes toward HIV among the non-medical working population in Japan. Knowledge may be needed to neutralize negative attitudes toward HIV, though people’s health consciousness may sometimes be a barrier. Further studies are needed to validate these findings and elucidate their mechanisms.

### Conclusion

More than one-third of individuals exhibited negative attitudes toward HIV infection. Knowledge is needed to neutralize this situation. Despite a relatively high level of knowledge overall, certain areas should be emphasized in the future. However, greater health concerns increase negative attitudes. Educational programs should balance knowledge with health consciousness for effective intervention.

## References

[1] Ha TH, Liu H, Li J, Nield J, Lu Z (2012) Psychometric assessment of scales measuring HIV public stigma, drug-use public stigma and fear of HIV infection among young adolescents and their parents. AIDS Care 24: 39–45. 21756072 10.1080/09540121.2011.592820 PMC3206131

[2] Stein JA, Li L (2008) Measuring HIV-related stigma among Chinese service providers: confirmatory factor analysis of a multidimensional scale. AIDS Behav 12: 789–795. 18064554 10.1007/s10461-007-9339-z PMC2700338

[3] Lieber E, Li L, Wu Z, Rotheram-Borus MJ, Guan J (2006) HIV/STD stigmatization fears as health-seeking barriers in China. AIDS Behav 10: 463–471. 16374668 10.1007/s10461-005-9047-5 PMC1553183

[4] Holzemer WL, Makoae LN, Greeff M, Dlamini PS, Kohi TW, et al (2009) Measuring HIV stigma for PLHAs and nurses over time in five African countries. SAHARA J 6: 76–82. 19936409 10.1080/17290376.2009.9724933 PMC3630506

[5] Nyblade LC (2006) Measuring HIV stigma: existing knowledge and gaps. Psychol Health Med 11: 335–345. 17130069 10.1080/13548500600595178

[6] Berger BE, Ferrans CE, Lashley FR (2001) Measuring stigma in people with HIV: psychometric assessment of the HIV stigma scale. Res Nurs Health 24: 518–529. 11746080 10.1002/nur.10011

[7] Hassan ZM, Wahsheh MA (2011) Knowledge and attitudes of Jordanian nurses towards patients with HIV/AIDS: findings from a nationwide survey. Issues Ment Health Nurs 32: 774–784. 22077750 10.3109/01612840.2011.610562

[8] Tee Y, Huang M (2009) Knowledge of HIV/AIDS and attitudes towards people living with HIV among the general staff of a public university in Malaysia. SAHARA J 6: 179–187. 20485857 10.1080/17290376.2009.9724946

[9] Dray-Spira R, Persoz A, Boufassa F, Gueguen A, Lert F, et al (2006) Employment loss following HIV infection in the era of highly active antiretroviral therapies. Eur J Public Health 16: 89–95. 16126745 10.1093/eurpub/cki153

[10] AIDS Action (1992) AIDS is everybody’s business: reaching people at work: programmes in Uganda, India and Zimbabwe. AIDS Action: 4–5. 12286003

[11] Liu Y, Canada K, Shi K, Corrigan P (2012) HIV-related stigma acting as predictors of unemployment of people living with HIV/AIDS. AIDS Care 24: 129–135. 21777074 10.1080/09540121.2011.596512

[12] Woods SP, Weber E, Weisz BM, Twamley EW, Grant I (2011) Prospective memory deficits are associated with unemployment in persons living with HIV infection. Rehabil Psychol 56: 77–84. 21401289 10.1037/a0022753 PMC3264430

[13] Reynolds GL, Fisher DG, Estrada AL, Trotter R (2000) Unemployment, drug use, and HIV risk among American Indian and Alaska Native drug users. Am Indian Alsk Native Ment Health Res 9: 17–32. 11279551 10.5820/aian.0901.2000.17

[14] Delpierre C, Cuzin L, Lauwers-Cances V, Datta GD, Berkman L, et al (2008) Unemployment as a risk factor for AIDS and death for HIV-infected patients in the era of highly active antiretroviral therapy. Sex Transm Infect 84: 183–186. 18192292 10.1136/sti.2007.027961

[15] Gilmour S, Li J, Shibuya K (2012) Projecting HIV transmission in Japan. PLoS One 7: e43473. 22916268 10.1371/journal.pone.0043473 PMC3423344

[16] Kuwahara T, Nakakura T, Oda S, Mori M, Uehira T, et al (2008) Problems in three Japanese drug users with Human Immunodeficiency Virus infection. J Med Invest 55: 156–160. 18319560 10.2152/jmi.55.156

[17] UNAIDS (2012) Progress reports submitted by countries. Ministry of Health LaW, Japan. Report to UNAIDS-HIV/AIDS trends in Japan, February 2012.

[18] UNAIDS (2003) Progress Report on the Global Response to the HIV/AIDS Epidemic, 2003. Available: http://www.unaids.org/en/media/unaids/contentassets/dataimport/topics/ungass2003/ungass_report_2003_en.pdf. Accessed 2013 June 15.

[19] Adebamowo CA, Ezeome ER, Ajuwon JA, Ogundiran TO (2002) Survey of the knowledge, attitude and practice of Nigerian surgery trainees to HIV-infected persons and AIDS patients. BMC Surg 2: 7. 12201903 10.1186/1471-2482-2-7 PMC126215

[20] Askarian M, Hashemi Z, Jaafari P, Assadian O (2006) Knowledge about HIV infection and attitude of nursing staff toward patients with AIDS in Iran. Infect Control Hosp Epidemiol 27: 48–53. 16418987 10.1086/500002

[21] Arigbede AO, Ogunrinde TJ, Okoje VN, Adeyemi BF (2011) HIV/AIDS and clinical dentistry: assessment of knowledge and attitude of patients attending a university dental centre. Niger J Med 20: 90–95. 21970267

[22] Kushimo OT, Akpan SG, Desalu I, Merah NA, Ilori IU (2007) Knowledge, attitude and practices of Nigerian anaesthetists in HIV infected surgical patients- a survey. Niger Postgrad Med J 14: 261–265. 17767215

[23] Qu B, Zhang Y, Guo H, Sun G (2010) Relationship between HIV/AIDS knowledge and attitude among student nurses: a structural equation model. AIDS Patient Care STDS 24: 59–63. 20113151 10.1089/apc.2009.0190

[24] Ryalat ST, Sawair FA, Shayyab MH, Amin WM (2011) The knowledge and attitude about HIV/AIDS among Jordanian dental students: (Clinical versus pre-clinical students) at the University of Jordan. BMC Res Notes 4: 191. 21676216 10.1186/1756-0500-4-191 PMC3127964

[25] Al-Rabeei NA, Dallak AM, Al-Awadi FG (2012) Knowledge, attitude and beliefs towards HIV/AIDS among students of health institutes in Sana’a city. East Mediterr Health J 18: 221–226. 22574474 10.26719/2012.18.3.221

[26] Ahmed SI, Hassali MA, Aziz NA (2009) An assessment of the knowledge, attitudes, and risk perceptions of pharmacy students regarding HIV/AIDS. Am J Pharm Educ 73: 15. 19513153 10.5688/aj730115 PMC2690879

[27] Agrawal HK, Rao RS, Chandrashekar S, Coulter JB (1999) Knowledge of and attitudes to HIV/AIDS of senior secondary school pupils and trainee teachers in Udupi District, Karnataka, India. Ann Trop Paediatr 19: 143–149. 10690254 10.1080/02724939992464

[28] Tung WC, Lu M, Cook DM (2012) HIV/AIDS Knowledge and Attitudes Among Chinese College Students in the US. J Immigr Minor Health. 10.1007/s10903-012-9716-1 22965498

[29] Genberg BL, Hlavka Z, Konda KA, Maman S, Chariyalertsak S, et al (2009) A comparison of HIV/AIDS-related stigma in four countries: negative attitudes and perceived acts of discrimination towards people living with HIV/AIDS. Soc Sci Med 68: 2279–2287. 19427086 10.1016/j.socscimed.2009.04.005 PMC4029331

[30] Ackerson LK, Ramanadhan S, Arya M, Viswanath K (2012) Social Disparities, Communication Inequalities, and HIV/AIDS-Related Knowledge and Attitudes in India. AIDS Behav 16: 2072–2081. 21870161 10.1007/s10461-011-0031-y

[31] Nagelkerke NJ, Jha P, de Vlas SJ, Korenromp EL, Moses S, et al (2002) Modelling HIV/AIDS epidemics in Botswana and India: impact of interventions to prevent transmission. Bull World Health Organ 80: 89–96. 11953786 PMC2567721

[32] Leclerc PM, Garenne M (2008) Commercial sex and HIV transmission in mature epidemics: a study of five African countries. Int J STD AIDS 19: 660–664. 18824616 10.1258/ijsa.2008.008099

[33] Boer H, Emons PA (2004) Accurate and inaccurate HIV transmission beliefs, stigmatizing and HIV protection motivation in northern Thailand. AIDS Care 16: 167–176. 14676023 10.1080/09540120410001641011

[34] Carr RL, Gramling LF (2004) Stigma: a health barrier for women with HIV/AIDS. J Assoc Nurses AIDS Care 15: 30–39. 10.1177/1055329003261981 15358923

[35] Earnshaw VA, Chaudoir SR (2009) From conceptualizing to measuring HIV stigma: a review of HIV stigma mechanism measures. AIDS Behav 13: 1160–1177. 19636699 10.1007/s10461-009-9593-3 PMC4511707

[36] Krasnik A, Wangel M (1990) AIDS and Danish adolescents–knowledge, attitudes, and behaviour relevant to the prevention of HIV-infection. Dan Med Bull 37: 275–279. 2357908

[37] Chen JQ, Dunne MP, Zhao DC (2004) HIV/AIDS prevention: knowledge, attitudes and education practices of secondary school health personnel in 14 cities of China. Asia Pac J Public Health 16: 9–14. 18839862 10.1177/101053950401600103

[38] Nur N (2012) Turkish school teachers’ knowledge and attitudes toward HIV/AIDS. Croat Med J 53: 271–277. 22661141 10.3325/cmj.2012.53.271 PMC3368290

[39] Lifson AR, Demissie W, Tadesse A, Ketema K, May R, et al (2012) HIV/AIDS stigma-associated attitudes in a rural Ethiopian community: characteristics, correlation with HIV knowledge and other factors, and implications for community intervention. BMC Int Health Hum Rights 12: 6. 22553906 10.1186/1472-698X-12-6 PMC3512528

[40] Korhonen T, Kylma J, Houtsonen J, Valimaki M, Suominen T (2012) University students’ knowledge of, and attitudes towards, hiv and AIDS, homosexuality and sexual risk behaviour: a questionnaire survey in two finnish universities. J Biosoc Sci 44: 661–675. 22739116 10.1017/S0021932012000338

[41] Cherutich P, Kaiser R, Galbraith J, Williamson J, Shiraishi RW, et al (2012) Lack of knowledge of HIV status a major barrier to HIV prevention, care and treatment efforts in Kenya: results from a nationally representative study. PLoS One 7: e36797. 22574226 10.1371/journal.pone.0036797 PMC3344943

[42] Ibrahim N, Rampal L, Jamil Z, Zain AM (2012) Effectiveness of peer-led education on knowledge, attitude and risk behavior practices related to HIV among students at a Malaysian public university–A randomized controlled trial. Prev Med 55: 505–510. 22982947 10.1016/j.ypmed.2012.09.003

[43] Gao X, Wu Y, Zhang Y, Zhang N, Tang J, et al (2012) Effectiveness of school-based education on HIV/AIDS knowledge, attitude, and behavior among secondary school students in Wuhan, China. PLoS One 7: e44881. 22970322 10.1371/journal.pone.0044881 PMC3436789

